# New Lamps for the Light Treatment

**Published:** 1901-10-05

**Authors:** 


### NEW LAMPS FOR THE LIGHT TREATMENT.

Various new forms of lamps are being devised for the application of the "light treatment," all of which seem to involve the principle of bringing the light much nearer to the patient than is the case with Finsen's lamp. In the gradual evolution of a machine, false steps are often made by adapting new means to old methods. The light first used in the treatment of lupus was that of the sun, and the apparatus was devised with the object of collecting and concentrating the somewhat scattered rays of ordinary sunlight. When, then, Finsen began to employ the electric light, he placed it at some distance from the patient, and collected its rays much as he had done those of the sun. In view, however, of the very rapid diminution of the intensity of light with every increase of distance from its source and the loss in its concentration, it is obvious that this must be a very wasteful proceeding. The closer the lamp can be brought to the point at which the light has to be applied the better, allowing room, of course, for the fluid by which its heat rays are to be absorbed, and this principle of putting the light in a box and bringing it as close as possible to the object is, we understand, the essence of the various new lamps which threaten speedily to displace those originally employed. In the lamp which Dr. Sequeira, of the London Hospital, has devised, the whole body of the lantern forms a water jacket, through which cold water is constantly circulating, so that heat is carried away not merely from between the lenses but from all round the arc light, which is so completely shut in that the lamp can be brought close to the patient without the latter running any risk or suffering any annoyance.

### THE SCRAPING OF LUPUS.

In all attempts to cure lupus by scraping there are two difficulties—one, that of making sure that the diseased patch is entirely removed ; the other, that of preventing the re-inoculation of the scraped surface by the material which is being removed. Dr. Herbert Snow [1] says that a method which he has employed for several years consists in scraping away as thoroughly as possible the soft-cell growth, and then, as soon as the bleeding has ceased, applying to the raw surface lint soaked in liniment of iodine. The latter is removed on the following morning. After treatment in this manner, he says, recurrence is most exceptional—in fact, never occurs except where a mucous membrane in close proximity, as that of the mouth or nose in facial cases, is also implicated. Dr. Snow had previously tried the use of iron lint—*i.e.*, lint soaked in liquor ferri perchloridi fort.—and this proved very efficient, but it had the disadvantage of occasionally causing an unsightly keloid scar, a drawback which does not follow the use of the iodine. The iron lint, however, has its place in the treatment of lupus when the nares, as so often happens, are implicated. Plugs left in after erosion immediately arrest all hæmorrhage, besides destroying residual growth.

[1] Brit. Med. Jour., Sept. 28.

